# Effects of the combination of biochar and organic fertilizer on soil properties and agronomic attributes of soybean (*Glycine max* L.)

**DOI:** 10.1371/journal.pone.0310221

**Published:** 2024-09-19

**Authors:** Marianus Evarist Ngui, Yong-Hong Lin, I-Lang Wei, Chia-Chung Wang, Ya-Zhen Xu, Ying-Hong Lin

**Affiliations:** 1 Department of Tropical Agriculture and International Cooperation, National Pingtung University of Science and Technology, Pingtung, Taiwan; 2 Department of Plant Industry, National Pingtung University of Science and Technology, Pingtung, Taiwan; 3 Department of Plant Industry, Soil and Fertilizer Laboratory, National Pingtung University of Science and Technology, Pingtung, Taiwan; 4 Department of Plant Medicine, Molecular Plant Medicine Laboratory, National Pingtung University of Science and Technology, Pingtung, Taiwan; Kobe University: Kobe Daigaku, JAPAN

## Abstract

This research aimed to investigate the impacts of a combination of rice husk biochar and organic fertilizer on the physical and chemical properties of soil, the population of soil bacteria, the relative chlorophyll content of leaves, the development of soybean root nodules, and yield components under strongly acid soil conditions. A greenhouse and pot experiment was designed using a randomize complete block design with factorial 2 × 3 treatments and three replications. The experimental treatments comprised two rates of biochar (35 and 70 g/pot) and three rates of organic fertilizer (70, 105, and 140 g/pot). After 100 days of amendment of strongly acidic soils, the results showed that application of treatments B35F70 and B70F140 increased soil pH by 16.80% compared to the control group (CK). On the other hand, treatments B35F140 and B70F105 resulted in an increase of soil electrical conductivity by 66.67% compared to CK. In addition, after 100 days of amendment with treatments B35F105, B35F105, B35F140, B70F105, B70F70, B70F70, and B35F140, organic matter, available phosphorous (P), potassium (K), calcium (Ca), magnesium (Mg), copper (Cu), and zinc (Zn), organic matter, available phosphorous (P), potassium (K), calcium (Ca), magnesium (Mg), copper (Cu), and zinc (Zn), significantly increased when compared to the control group (CK). Treatment B35F140 increased relative leaf chlorophyll content and soybean seed weight per plant by 60.76% and 100.56%, respectively when compared to the CK. Furthermore, treatment B35F70 produced 125% more root nodules than CK. Moreover, each amended strongly acid soil resulted with a significant upsurge in total soil bacteria compared to the CK. Overall, statistics proved that a combination of biochar and organic fertilizer improved soil properties and soybean agronomic attributes.

## Introduction

Soybeans are important in Taiwan due to their high protein content, potential as a non-cholesterol oil, and significance in the livestock industry as a potential raw material for making animal feeds. The beans are rich in eight essential amino acids, vitamins, flavonoids, and polysaccharides required by the human body [1]. The quality of soybean protein is comparable to animal proteins from meat, milk, and eggs [2]. According to [3], consumption of soybean protein may reduce the risk of breast and prostate cancer, benefit the kidneys, alleviate diabetes, lower blood pressure, and relieve depression. Moreover, the use of soybean protein has been found to inhibit fat accumulation and increase its metabolism [4]. In climate-change affected areas, soybeans proved their ability to grow under different cropping and ecological systems [5,6]. By adopting the appropriate agronomic practices, soybean plants have produced a reasonable yield under environmental stresses [7,8].

High crop yields are a result of advancements in modern agricultural technology, particularly the development of inorganic fertilizers, which modern agriculture heavily relies on [9]. However, aside from climate change, the degradation of soil properties and the loss of arable land are the two primary challenges that modern agriculture faces [10,11]. Most of these problems are associated with the excessive use of chemical fertilizers in the pursuit of increasing crop production [12]. It has been globally recognized that the extensive application of inorganic fertilizer leads to the loss of organic matter, a decrease in soil pH, and ultimately, a decline in soil fertility [13,14]. Accordingly, the low-fertility soils cannot produce the expected output of agricultural crops, which threatens food security [15]. Nevertheless, due to advancements in agricultural technology, soil pH can be corrected to a moderate acidic or near neutral condition, which supports the optimal growth and productivity of most crops. Applying lime is a common management technique used to correct acid soils in agricultural land, but chemical lime does not have long-lasting effects on agricultural soils. Moreover, the effects of liming wear off quickly, which can lead to the re-acidification of the soil. Additionally, industrial lime is considered a source of soil pollution due to the introduction of heavy metals into the soil [16].

Understanding soil pH is essential for successful crop production. The pH of the soil determines the acidity or alkalinity of the soil. In addition, the pH of soil directly affects the quantity and availability of essential plant nutrients [17]. Plant micro-nutrients, boron (B), copper (Cu), manganese (Mn), iron (Fe), and zinc (Zn) are more readily available in soils with a pH below 5.5, while plant macro-nutrients, nitrogen (N), phosphorous (P), potassium (K), calcium (Ca), and magnesium (Mg) are more readily available in soils with a pH above 5.5. Additionally, soil pH significantly influences the activity of soil microorganisms, including worms, fungi, and bacteria, which play a crucial role in breaking down organic matter and releasing plant nutrients into the soil[18]. It is important to note that strongly acidic soils are characterized by nutrient deficiency and the toxicity of metals such as aluminium (Al) and iron (Fe) [19]. Aluminium is particularly harmful to plant roots [20]. It inhibits root growth and negatively impacts water and nutrient uptake in plants [21].

Recently, recycling and exploiting agricultural wastes as soil conditioners has received great attention, associating with crop production sustainability [22–24]. The pyrolysis of agricultural crop residues to produce biochar, a material that is primarily alkaline and rich in essential plant nutrients, shows promise as a sustainable soil fertility management technology. Biochar is produced through the pyrolysis process of agricultural crop residues at temperatures ranging from 300 to 600°C [25]. Due to its high pH and the presence of carbonates and organic anions from acid functional groups [26], the use of biochar is compelling as an alternative to synthetic chemical lime for amending strongly acid soil and improving soil fertility [27]. In addition, biochar can enhance the pH buffering capacity of the soil, thereby preventing the re-acidification of amended soils [28]. Furthermore, the application of both biochar and organic fertilizer in agricultural soils offers valuable environmental benefits [29–31]. Moreover, the combined use of biochar and organic fertilizer provides significant support in achieving Sustainable Development Goals (SDGs) 1 (no poverty), 2 (zero hunger), 3 (good health and well-being), 7 (affordable and clean energy), 12 (responsible consumption and production), 13 (climate action), 15 (life on land), and 17 (partnership for the goals).

**Fig 1:**
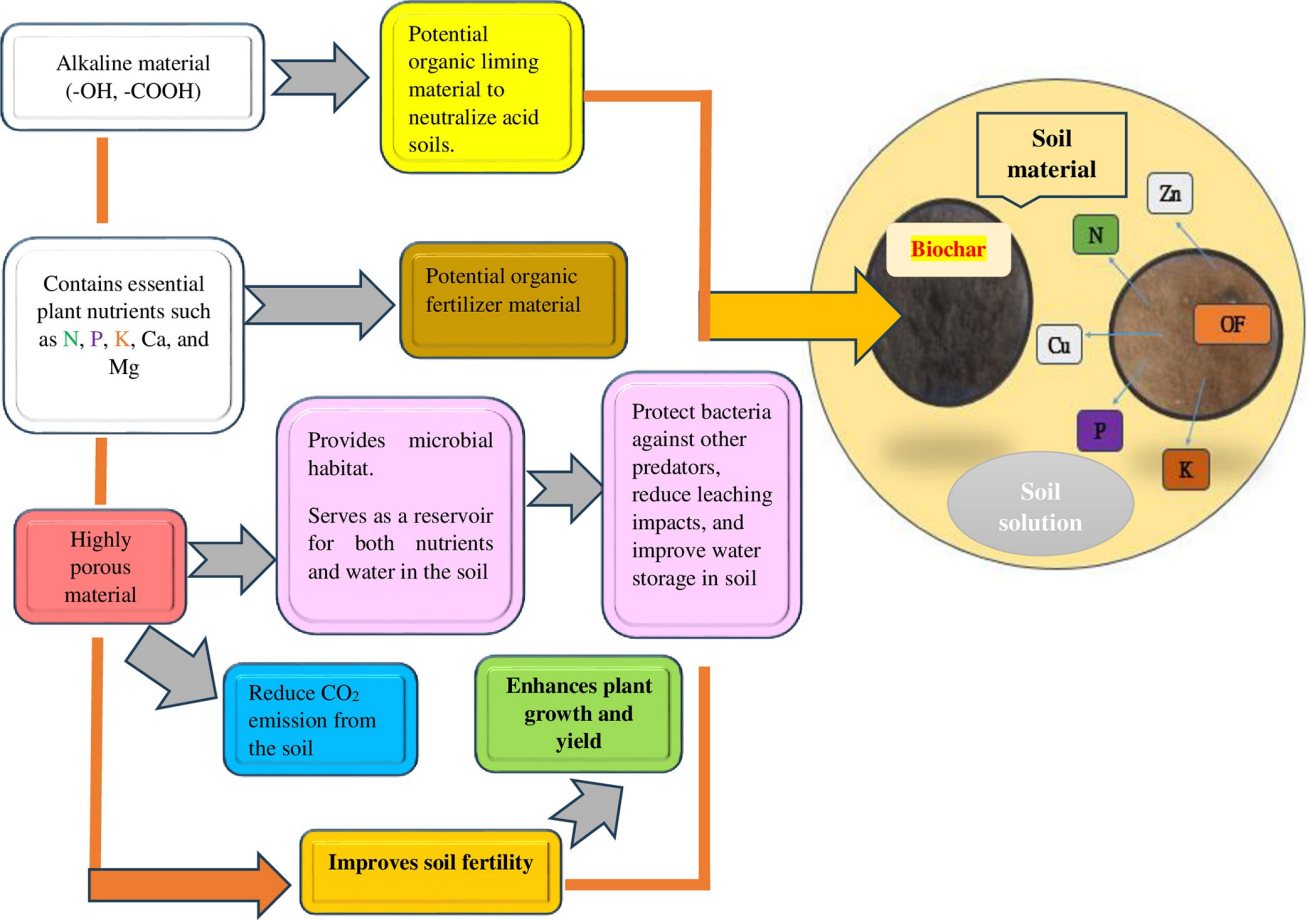
The conceptual mechanism of the interaction between biochar and organic fertilizer (OF) in soil. When soil organic matter contained in organic fertilizer is mineralized by soil bacteria, N, P, K, Cu, and Zn nutrients are released into the soil solution. Subsequently, the dissolved minerals are adsorbed by the biochar surface due to the numerous pores that cover its surface. This enhances the retention of various nutrients in the soil-biochar-organic fertilizer interface for a long period to benefit plants during the development.

According to [32], biochar is noted for possessing a number of advantageous qualities that make it a potential option in agricultural soils (Fig 1). However, [33] discovered that the sole application of biochar is not sufficient to achieve crop productivity. This is because the effectiveness of biochar depends on various factors, including the type of feedstock used to produce biochar, the pyrolysis temperature, and the type of soil that is amended with biochar. Similarly, the application of organic fertilizer is known to enhance soil quality, crop growth, and yield by supplying essential nutrients such as N, P, and K [34]. This is achieved through the mineralization process, which is mostly carried out by the soil microorganisms, particularly the bacteria, fungi, and earthworms. Additionally, the application of organic fertilizer is reported to supply essential crop nutrients in soils such as Ca, Mg, Zn, Cu, Mn, and Fe. The mechanisms behind this, is due to improved nutrients synchrony [35] and priming effect [36]. Moreover, soil water and nutrient holding capacity, soil aggregation and structure that allow easy air, water, and roots movement in soil are enhanced through the incorporation of organic fertilizers into the soil [37]. In addition, both biochar and organic fertilizer have physical properties which enhance the storage of both water and nutrients for a long-time during crop growth, thus reducing the leaching of nutrients. Moreover, the effects of biochar on soil can persist for up to four years since its first application [38].

Therefore, the current study is intended to investigate the effectiveness of a combination of rice husk biochar and organic fertilizer on soil characteristics and soybean yield components under strongly acidic soil conditions. Due to the limited availability of scientific research on the effects of combining biochar and organic fertilizer on soil properties and legume crop yields in strongly acidic soil, this study aims to offer valuable insights for soybean farmers, particularly in Taiwan. The goal was to help them increase their on-farm soybean productivity, which is currently limited by the unfavourable conditions of strongly acidic soils. However, based on our awareness of the already-conducted studies, this investigation is the first scientific report that details the impacts of a combination of rice husk biochar and a mixture of different types of organic fertilizers on soil properties and soybean productivity in a strongly acidic soil.

## Materials and methods

### Soil, biochar, organic fertilizer, and soybean seed variety

An experimental soil sample was collected from Taiwu Township, Pingtung, Taiwan. Rice husk biochar was selected in this study due to its potential availability among rice farmers in the research area (NPUST, Taiwan). During biochar production, the rice husks were subjected to a 24-hour pyrolysis process at a temperature of 600°C. The pyrolysis process, which includes four dissimilar stages, namely, moisture evolution, hemicellulose decomposition, cellulose decomposition, and lignin decomposition, was conducted at the Sin-Fong Rice Milling Factory located in Pingtung, Taiwan. The organic fertilizer was made up of a mixture of sawdust, soybean residues, and manure from cattle, chickens, and pigs. The Nancho Farmer’s Association in Pingtung, Taiwan, was responsible for organic fertilizer making. The *Tainan 10 soybean seed variety* used in the experiment was sourced from the Tainan District of Agricultural Research and Extension Station.

### Soil, biochar, and organic fertilizer analysis

A composite soil sample (0–20 cm deep) was collected prior to amendment for the purpose of analyzing its physical, chemical, and biological characteristics. The soil sample was allowed to air dry, ground, and then sieved using a 2-mm mesh. The soil organic matter content was determined using the Walkley-Black method [39]. Soil pH in water at a ratio of 1:5 was measured potentiometrically using an electrode meter [40]. According to [41,42], soil available phosphorous (P), potassium (K), calcium (Ca), magnesium (Mg), copper (Cu), zinc (Zn), iron (Fe), manganese (Mn), and sodium (Na) were analyzed by inductively coupled plasma atomic mass spectrometry (ICP-AMS) at wavelengths of 213.62 nm, 766.49 nm, 317.93 nm, 279.81 nm, 324.75 nm, 213.86 nm, 259.94 nm, 257.61 nm, and 589.59 nm, respectively. The analyzed soil physico-chemical properties were as follows: pH 5.13, organic matter 4.04%, available P 333.63 mg/kg, K 64.44 mg/kg, Ca 1,198.24 mg/kg, Mg 24.29 mg/kg, Cu 0.51 mg/kg, and Zn 2.58 mg/kg. The initial population of soil bacteria was 6.81 log CFU/g. The soil textural class was clay loam, comprised of 32% sand, 30% silt, and 38% clay. The biochar pH was 9.89, with available P (4,455.47 mg/kg), K (10, 605.53 mg/kg), Ca (2,016.42 mg/kg), and Mg (1,112.65 mg/kg). The physico-chemical properties of the organic fertilizer were as follows: pH 8.22, organic matter 61%, total N 2.55%, P 2.3%, and K, 2.0%.

### Experimental duration and design

The experiment was conducted from September 2023 to February 2024. The study utilized a randomized complete block design (RCDB) with factorial 2 × 3 treatments, replicated three times, using pots as experimental units. The experiment was conducted in the greenhouse of the Department of Plant Industry at the National Pingtung University of Science and Technology. The experimental treatments consisted of two rates of biochar (35 and 70 g/pot) and three rates of organic fertilizer (70, 105, and 140 g/pot) (Table 1). The decision on biochar application rates considered the mutual benefits of organic fertilizer. Because previous studies [43–46] employed high rates of biochar, we chose to utilize low rates, bearing in mind that the organic fertilizer will also impact soil characteristics through the release of essential nutrients as biochar does to soil, encouraging water and nutrient holding capacity, ameliorating soil pH, and promoting soil microbial growth. The choice of application rate of organic fertilizer was decided based on the fertilizer requirements recommended by the breeder of the Tainan 10 soybean seed variety, which were 40–60 kg of N per hectare (Ha), 60 kg of P_2_O_5_ per ha, and 60 kg of K_2_O per ha. In total, there were twenty-one experimental pots, each measuring 30 cm in diameter and 30 cm in height. To maintain consistent temperature conditions, an automatic rotating fan system was employed, maintaining a temperature of 25°C at night and 30°C during the day inside the greenhouse.

**Table 1:** Biochar and organic fertilizer treatment details.

Treatments	Application rates (g/10.5 kg of soil in each pot)
CK	0 g of Biochar + 0 g of Organic fertilizer
B35F70	35 g of Biochar + 70 g of Organic fertilizer
B35F105	35 g of Biochar + 105 g of Organic fertilizer
B35F140	35 g of Biochar + 140 g of Organic fertilizer
B70F70	70 g of Biochar + 70 g of Organic fertilizer
B70F105	70 g of Biochar + 105 g of Organic fertilizer
B70F140	70 g of Biochar + 140 g of Organic fertilizer

CK represents un-amended soil. Numbers preceding letters B and F letters represent grams of applied biochar and organic fertilizer, respectively.

### Experimental procedures

**Fig 2:**
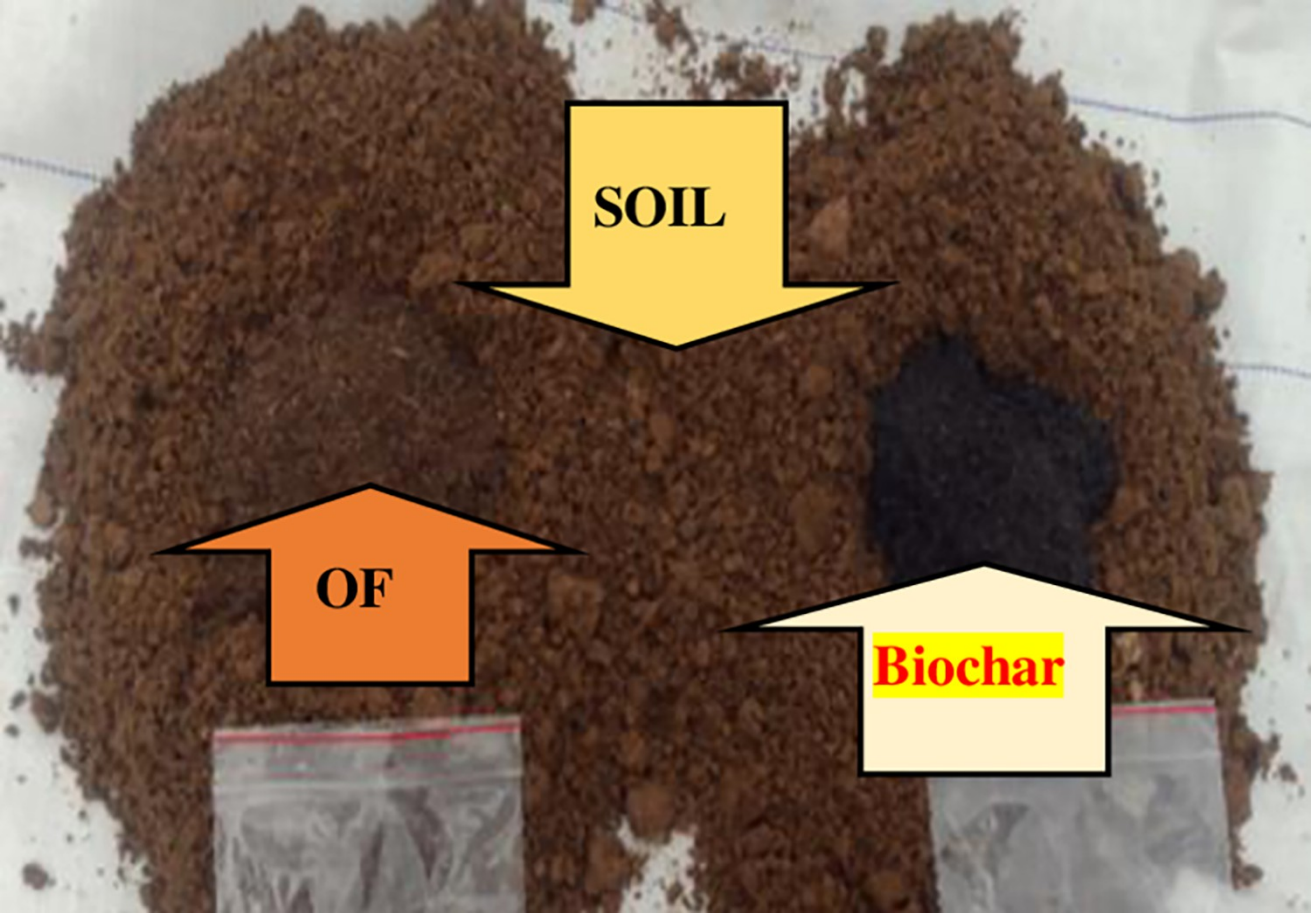
Preparation of combined soil containing Organic fertilizer (OF), acid soil (SOIL) and biochar.

**Fig 3:**
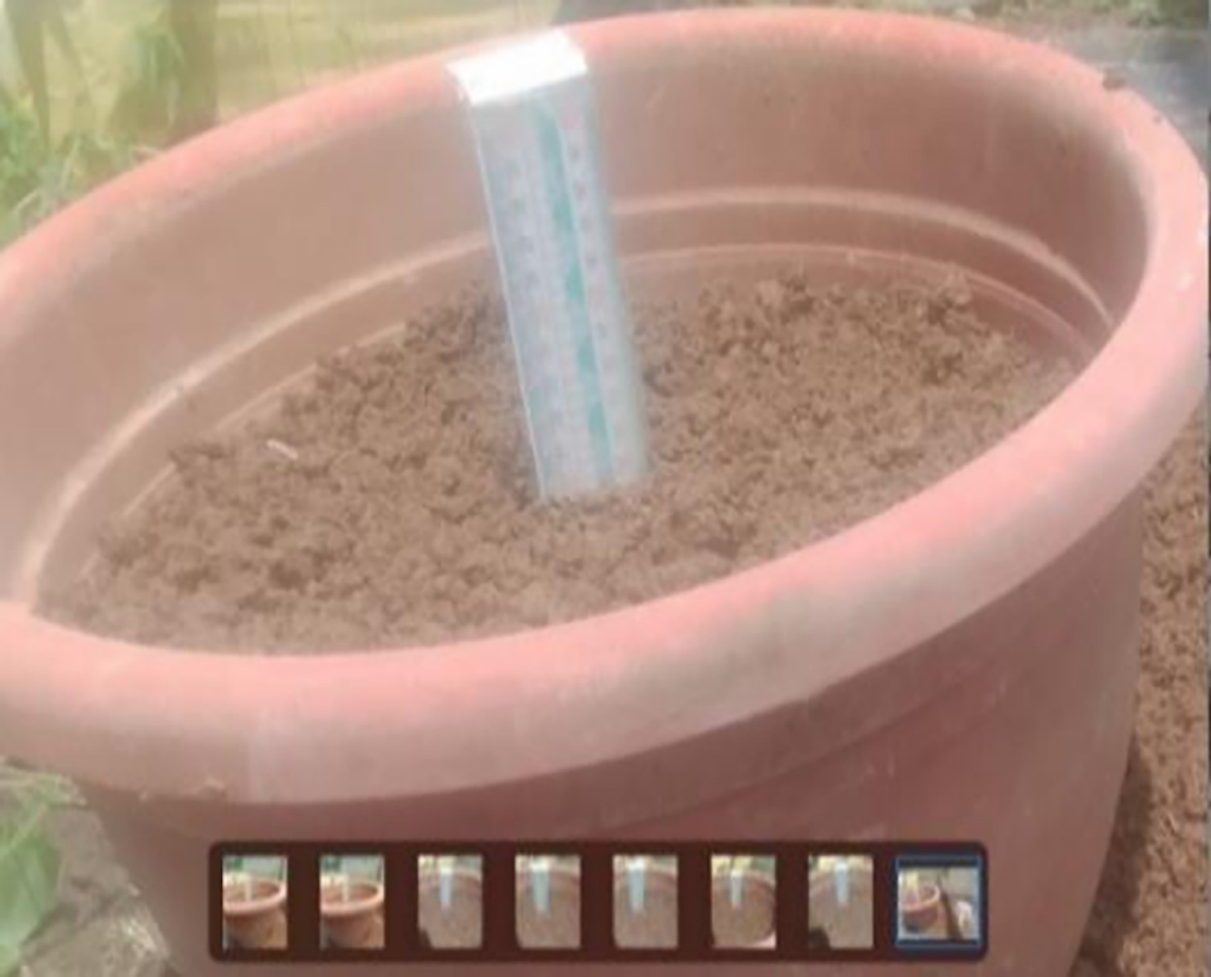
Amalgamated soil deposited in experimental pot.

A total of 10.5 kg of experimental soil was thoroughly mixed with biochar and organic fertilizer (Fig 2) and deposited in each pot with dimensions of 30 cm in diameter and 30 cm in height. The volume of soil deposited in each pot occupied a height of 20 cm (Fig 3). After the soil amendment, the amended soil was allowed to settle for 14 days before planting soybean seeds. This allowed for further incorporation of the biochar and organic fertilizer into the soil. No water was added to the soil combined with biochar and organic fertilizer during the 14-day period because the soil was already moist before the amendment. On the fifteenth day, seven soybean seeds were planted in each pot, spaced 5 cm apart and at a depth of 2.5 cm. After 7 to 10 days, when germination and seedling emergence, occurred, the soybean plants were thinned to two per pot. In total, 42 soybean plants were kept for observation. Once the seeds sprouted, each pot’s plants were irrigated. Soybean plants in each pot were irrigated with 400 milliliters (mL) of tap water during the vegetative growth phase. The frequency of irrigation was decided based on the readings obtained from the tensiometer, which was applied to monitor the moisture condition of the experimental soil. Irrigation was done when the tensiometer read 30 Kpa. During the flowering and pod filling stages, each pot was supplied with 500 and 600 mL of tap water, respectively. At the maturity stage, the amount of water supplied per pot was reduced to 300 mL. Throughout the growth period of the soybean plants, pests and weeds were managed.

### Data sampling and collection

To estimate the relative leaf chlorophyll content, a potable chlorophyll meter, SPAD-502 (Konica-Minolta, Tokyo), was utilized. The SPAD values were measured on three leaves of both plants in a pot, specifically the marked fourth trifoliate, and the results were averaged. Counts and measurements were conducted on both plants for soybean yield components (pod numbers, pod dry weight, number of seeds per plant, and seed weight per plant), and the average was calculated as well. Root nodules were counted after the soybean harvest. 100 days after amendment of strongly acid soil by using a combination of biochar and organic fertilizer, rhizosphere soil samples (0–20 cm deep) from each pot were collected for analysis of physical, chemical, and biological properties. We analyzed soil physico-chemical properties after 100 days of amendment due to the fact that organic fertilizers gradually release nutrients into soils, thus taking some time to achieve the optimal quantity of nutrients to support productivity of crop. To determine the quantity of bacteria in the soils, the soil dilution method was employed. 1 g of rhizosphere soil sample was taken from each pot and mixed with 9 mL of sterile distilled water in a 15 mL centrifuge tube. After one minute of stirring, soil solutions were subjected to 10× serial dilutions. Then 200 μL of the resulting diluted soil suspension were pipetted and placed on a petri dish containing potato dextrose agar (PDA) (20% potato extract, 2% dextrose, and 2% agar). The soil sample solution was spread evenly in each petri dish and then incubated at 25°C. The number of colonies formed after the first 72 hours was counted to determine the initial bacterial count of the soil [47].

### Data analysis

SAS version 9.4 (TS1M6, Cary, NC, USA) was utilized to conduct an analysis of variance (ANOVA) to compare the means. The least significant difference test at a significance level of p<0.05 was employed to identify any significant differences among the treatments. For data regarding the population of soil bacteria, SPSS version 20 (IBM, NY, USA) was employed, and Turkey’s test was used to assess the significant differences between the treatments at a significance level of p<0.05. Graphs were constructed using Excel software (Microsoft Office Excel, 2016) based on the means developed from three repetitions per treatment.

## Results

### Changes in soil pH, electrical conductivity (EC), and organic matter content

After 100 days of amendment of strongly acid soil, soil pH significantly increased in all amended soils compared to the CK. Treatments B35F70 and B70F140 increased the soil pH to 5.77 (Table 2). This change represents an increase of 7.25% and 16.80% compared to treatments B70F105 and CK, respectively. The changes in soil pH in treatments B35F105 and B70F105 were not significantly different from one another. Contrary to changes in soil pH in other amended soils, only treatment B70F105 resulted in a low change in soil pH and was still strongly acidic. Similarly, amendment of strongly acidic soil by using a combination of biochar and organic fertilizer resulted in a significant effect on soil electrical conductivity (EC). Each amended soil showed a significant increase in EC compared to the CK (Table 2). Furthermore, after 100 days of amendment, the lowest *SOM* content was found in treatment B35F140 (Table 2). It was lower by 2.67% than the CK. Conversely, treatment B35F105 resulted in the highest percentage of organic matter content, surpassing CK and B35F140 by 63.29% and 67.56%, respectively. Following the amendment of strongly acidic soils by using various rates of biochar and organic fertilizer, the percentage of soil organic matter (SOM) in the amended soil varied too (Table 2). Compared to CK, soil organic matter in the amended soils was significantly higher. Soil organic matter content in treatments B35F140, B70F70, and B70F140 was higher by 19.80%, 23.51%, and 20.79%, respectively, compared to the CK. Similarly, the three afore-mentioned treatments resulted in a high *SOM* of 8.28%, 9.91%, and 7.73% in comparison with treatments B35F70, B35F105, and B70F105, respectively. On the other hand, soils in CK had a low soil organic matter of 19.04% compared to treatment B35F70.

**Table 2:** Soil pH, electrical conductivity (EC), organic matter, available P, Ca, K, and Mg after 100 days of amendment of strongly acidic soil by using a combination of biochar and organic fertilizer.

Treatments	Soil pH (1:5 soil: water)	EC (mS/cm)	OM (%)	P (mg/kg)	Ca (mg/kg)	K (mg/kg)	Mg (mg/kg)
CK	4.94^a^	0.06^a^	3.46^a^	469.05^ab^	846.36^ab^	100.57^a^	71.81^a^
B35F70	5.77^c^	0.08^b^	4.79^ab^	771.88^b^	1,239.38^b^	229.37^b^	157.52^b^
B35F105	5.62^bc^	0.08^b^	5.65^b^	781.22^b^	1,153.32^ab^	307.07^c^	150.00^b^
B35F140	5.74^c^	0.10^b^	3.37^a^	661.25^ab^	1,182.34^ab^	316.21^c^	141.70^b^
B70F70	5.64^c^	0.09^b^	4.82^ab^	628.65^ab^	1,104.53^ab^	261.69^bc^	159.76^b^
B70F105	5.38^b^	0.10^b^	5.03^b^	398.95^a^	841.17^a^	305.44^c^	139.98^b^
B70F140	5.77^c^	0.08^b^	5.63^b^	628.35^ab^	1,143.14^ab^	270.93^bc^	148.50^b^

Means with the same superscript lowercase letter within the column do not significantly differ at (*p< 0*.*05*) according to the least significant difference (LSD). CK represents un-amended soil. Numbers preceding letters B and F represent grams of applied biochar and organic fertilizer, respectively.

### Soil available P, K, Ca, Mg, Cu, Zn, Fe, Mn, and Na

Soil available P significantly increased following the amendment of strongly acidic soil by using treatments B35F70 and B35F105. Soil amended with treatment B35F70 showed an increase in available phosphorous by 64.56% and 93.48% when compared to treatment CK and B70F105, respectively. Similarly, treatment B35F105 significantly increased available P by 66.55% and 95.82% in comparison to treatment CK and B70F105, respectively. However, there was no significant difference in available P among treatment CK, B35F140, B70F70, and B70F140. Nonetheless, treatments B35F140, B70F70, and B70F140 showed elevated available P by 40.98%, 34.03%, and 33.96% over the CK treatment (Table 2). The amendment of strongly acidic soil by using a combination of biochar and organic fertilizer resulted in an increase in available calcium. However, based on the soil texture class of experimental soil, clay loam soils are known to naturally contain a high level of soil calcium. Our findings indicated that only soil amended with treatment B35F70 exhibited a significant increase in available calcium by 47.34% when compared to treatment B70F105 (Table 2). Moreover, our results showed a significant increase in the availability of potassium in the amended soils. Treatment B35F140 was among the treatments that exhibited the highest concentration of available potassium. However, the available potassium in the said treatment did not differ significantly from the available potassium in soils amended with treatments B35F105, B70F105, B70F70, and B70F140. In the application of treatments B35F105, B35F140, and B70F105, the available potassium increased by 205.33%, 214.42%, and 203.71%, respectively, compared to the available potassium in the CK. Similarly, when comparing the former three treatments with treatment B35F70, there was an increase in available potassium by 37.86%, 33.88%, and 33.16%, respectively. In addition, the available potassium in treatments B35F70, B70F70, and B70F140 did not significantly differ from each other. The lowest amount of available potassium in the soil was found in treatment CK (Table 2). Moreover, positive effects as a result of amendment of strongly acid soil with a combination of biochar and organic fertilizer observed on the available magnesium in the soil. Each rate of a combination of biochar and organic fertilizer significantly increased soil available magnesium compared to the CK (Table 2).

Table 3 showed that the application of treatment B35F105 resulted in a significant high concentration of available Fe by 35.94% and 44.19% compared to CK and B35F140 treatments. However, the former treatment was significantly different compared to the available Fe in treatments CK, B35F140, B70F105, and B70F140. The lowest concentration of available Fe was found in soil amended with treatment B35F140. Moreover, the available Fe in the latter treatment did not statistically differ from that of CK, B70F105, and B70F140. Contrary to the findings of available Fe, the application of treatment B35F140 resulted in the highest concentration of available Cu. After 100 days of strongly acidic soil, soil available copper significantly increased in treatments B70F70 and B70F105 when compared to the CK, B35F70, and B35F105 treatments (Table 3). For the available Mn, treatment B70F105 resulted in the highest concentration of available manganese by 71.87%, 220.53%, and 272% in comparison to treatments CK, B35F70, and B70F140, respectively. However, the available Mn in treatments B35F105, B35F140, and B70F70 did not statistically differ from each other. The lowest concentration of available Mn was found in the application of treatment B70F140. Contrary to the findings of available copper, the amendment of strongly acidic soil through the application of treatments B35F70, B35F105, and B35F140 resulted in a significant increase in soil zinc availability in comparison to CK. In addition, soil available zinc in soils amended with treatments B70F70 and B70F105 significantly differed compared to CK. However, application of treatment B70F140 resulted in an increase in soil available zinc which did not significantly differ from treatments CK, B35F70, and B70F70. The amendment of strongly acid soil through treatment B35F140 resulted in a significant increase in available zinc by 52.28% and 97.26%, respectively, when compared to treatment B70F140 and CK (Table 3). Furthermore, the aamendment of strongly acidic soil through different combinations of biochar and organic fertilizer resulted in a significant difference in the available Na in the soil compared with the CK treatment (Table 3). In addition, we found that there was a linear regression relationship (R^2^ = 0.3728) between available Na and soil electric conductivity (Fig 6E).

**Table 3:** Soil available Ca, Fe, Cu, Mn, Zn, and Na after 100 days of amendment of strongly acidic soil by using a combination of biochar and organic fertilizer.

Treatments	Available trace elements				
	Fe (mg/kg)	Cu (mg/kg)	Mn (mg/kg)	Zn (mg/kg)	Na (mg/kg)
CK	181.02^ab^	0.87^a^	2.54^a^	4.74^a^	35.17^a^
B35F70	219.47^bc^	1.05^a^	4.87^ab^	7.44^bc^	88.76^c^
B35F105	246.07^c^	0.97^a^	6.95^bc^	9.20^c^	67.09^b^
B35F140	170.65^a^	1.25^ab^	5.43^abc^	9.35^c^	72.15^bc^
B70F70	221.47^bc^	1.94^b^	6.26^bc^	8.35^bc^	110.01^d^
B70F105	200.40^ab^	1.88^b^	8.37^c^	8.95^c^	80.19^bc^
B70F140	180.35^ab^	1.28^ab^	2.25^a^	6.14^ab^	78.43^bc^

Means with the same superscript lowercase letter within the column do not significantly differ at (*p< 0*.*05*) according to the least significant difference (LSD). CK represents un-amended soil. Numbers preceding letters B and F represent grams of applied biochar and organic fertilizer, respectively.

### Total population of soil microorganisms

**Fig 4:**
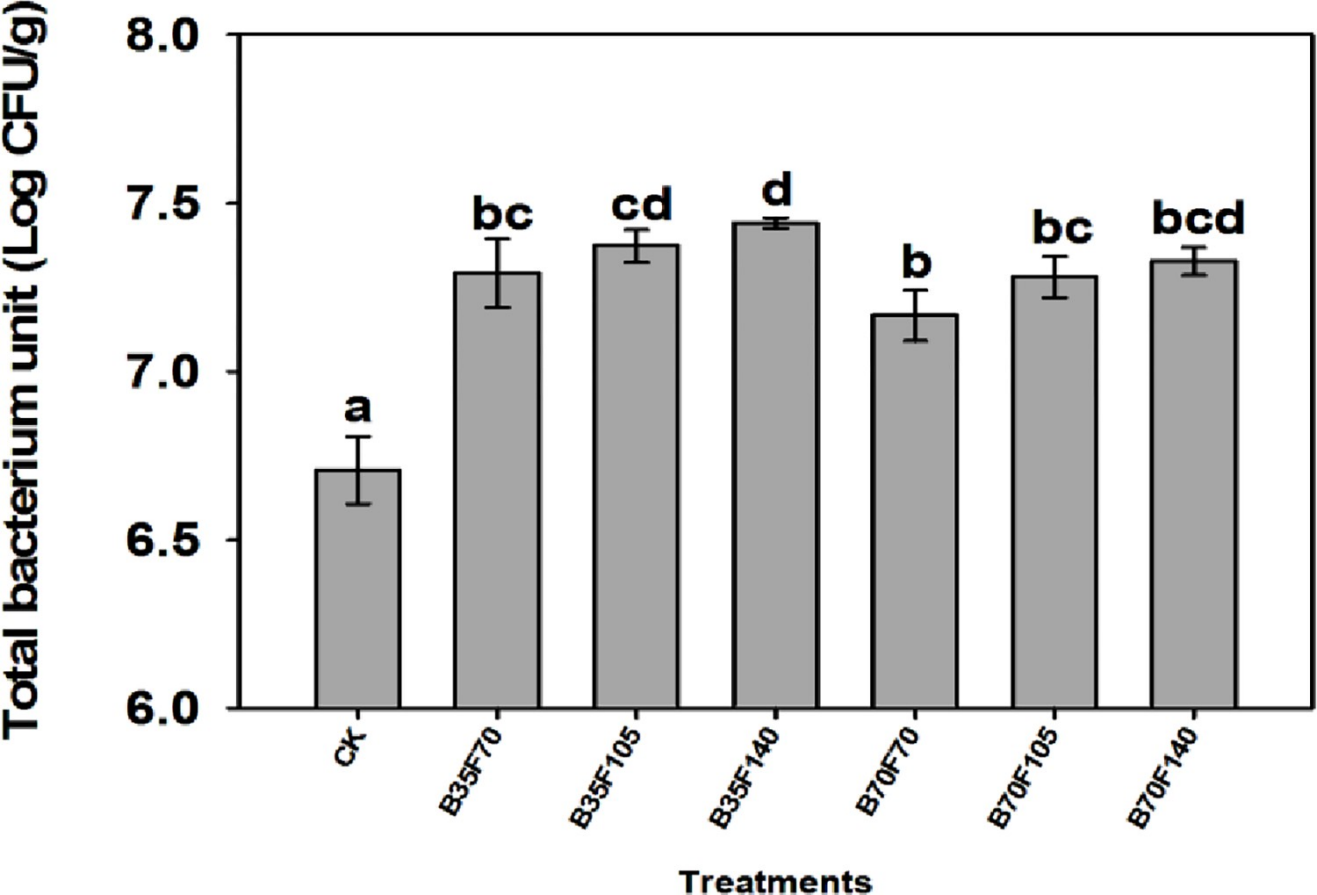
Total soil bacterial population per treatment after 100 days of amendment of strongly acidic soil. Bars sharing the same lower-case letters are not statistically significant, as determined by Turkeys test at a significance level of p< 0.05. Numbers preceding the letters B and F indicate grams of biochar and organic fertilizer applied, respectively. CK represents un-amended soil.

The study has revealed the efficacy of combining biochar and organic fertilizer to promote the growth of soil-borne bacteria. In comparison to the control group (CK), the total population of soil bacteria was significantly higher in all amended soils (Fig 4). Treatment B35F140 among the amended soils exhibited the highest population of soil bacteria, while treatment B70F70 had a slightly lower population compared to the other amended soils. Additionally, the results indicated that treatment B35F140 resulted in a more noticeable proliferation of soil bacteria compared to treatment B70F140. The control group (CK) showed the lowest proliferation of soil bacteria. However, in some petri dishes, there were observations of mycelium structures, indicating a small amount of fungal growth. Therefore, only the bacterial colonies were considered in numerous petri dishes.

### Average number of root nodules

**Fig 5:**
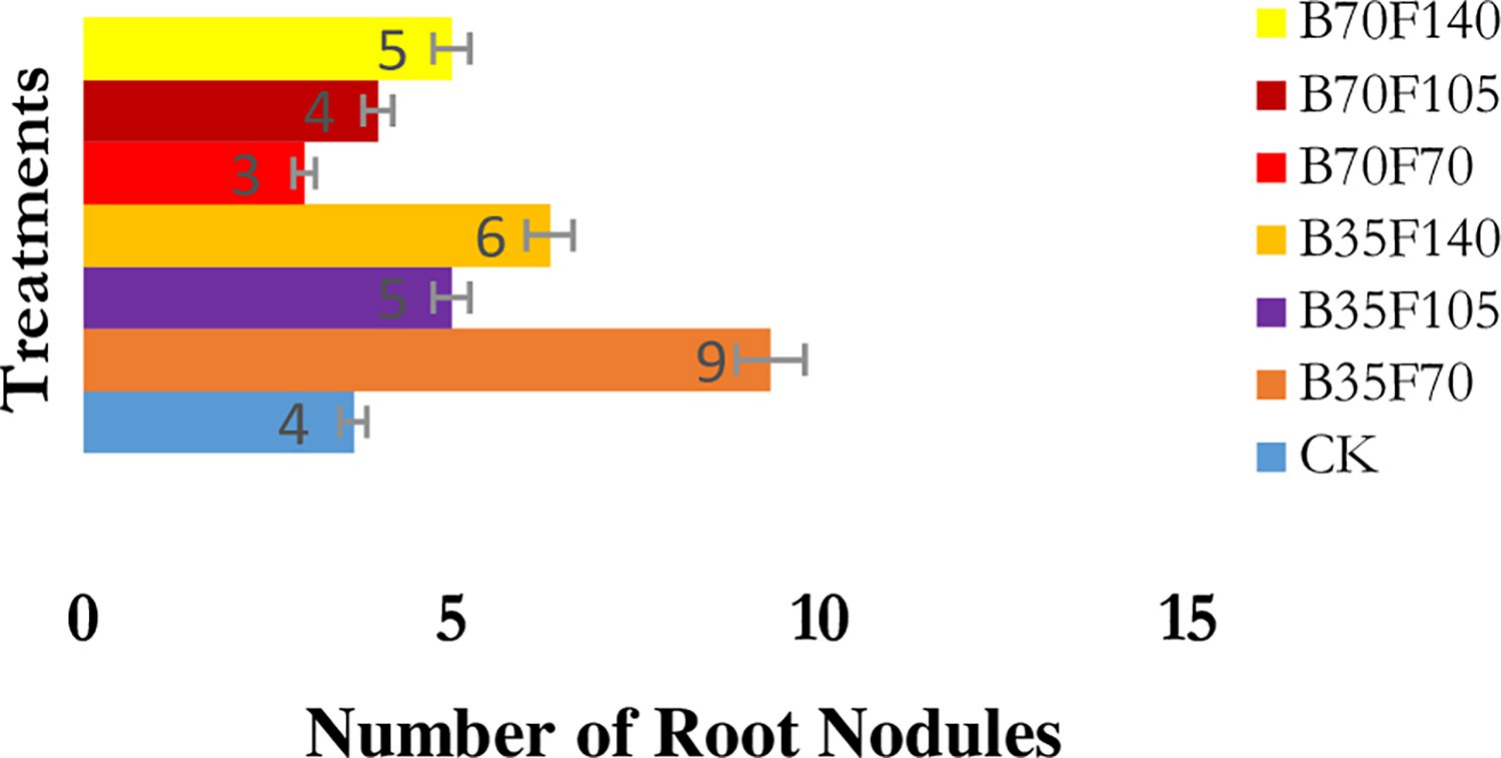
Average number of soybean root nodule per plant. Numbers preceding the letters B and F represent grams of applied biochar and organic fertilizer, respectively. CK represents un-amended soil.

Fig 5 displays the results of average soybean root nodules following the application of a combination of biochar and organic fertilizer. Soybean plants treated with B35F70, B35F140, and B70F140 exhibited a higher number of root nodules compared to those fertilized with other treatments. Root nodules on soybean plants grown under treatment B70F70 were the fewest by 25% compared to CK soybean plants. Compared to treatments B35F70 and CK, the former treatment had more root nodules by 125% than the latter treatment.

### SPAD values

SPAD values in soybean plants were assessed during the vegetative, flowering, and pod-filling stages. The study revealed a significant increase in relative leaf chlorophyll content in soybean plants cultivated in the amended soils compared to those in the CK soil group (Table 4). During the sixth week after planting soybeans, when pod filling took place, SPAD values in the application of treatments B35F70 and B70F70 were not significantly different. A similar trend was seen when treatment B35F105 was compared to B70F105 and B70F140. The highest SPAD values were determined following the amendment of strongly acidic soil by application of treatment B35F140, while the lowest were measured on soybean plant leaves cultivated under the control soil group (CK).

**Table 4:** Average SPAD values in application of combination of biochar and organic fertilizer.

Treatments	Vegetative stage	Flowering stage	Pod filling stage
	4 WAS	5 WAS	6 WAS
	**SPAD Values**		
CK	25.3^a^	28.0^a^	23.7^a^
B35F70	28.2^bc^	34.0^b^	33.2^bc^
B35F105	28.4^bc^	36.9^c^	36.8^cd^
B35F140	29.5^c^	37.4^c^	38.1^d^
B70F70	26.4^ab^	32.3^b^	31.0^b^
B70F105	28.7^c^	37.0^c^	37.0^cd^
B70F140	30.1^c^	36.1^c^	37.3^cd^

Means with the same superscript lower-case letter within the column do not significantly differ at (*p< 0*.*05*) according to the least significant difference (LSD). CK represents un-amended soil. SPAD represents Soil and Plant Analysis Development, and WAS represents Weeks after sowing. Numbers preceding letters B and F represent grams of applied biochar and organic fertilizer, respectively.

## Average of soybean yield components

After the harvest of soybeans, which was done after 98 days since the planting of soybean seeds, soybean yield components were assessed (Table 5). In every soybean plant grown on amended soil with varying amounts of combination of biochar and organic fertilizer, there was a significant different in the number of pods compared to the CK. In comparison to treatments B70F140 and CK, application of treatment B35F140 resulted in an increase of 11.09% and 105.91% of pods per plant, respectively. In contrast to treatment B35F105, adding 35 g of biochar to treatment B70F105 resulted in a 11.11% increase in pod number. On the other hand, compared to treatment B70F70, application of B35F70 increased the number of pods by 17.80%. Similar trends were seen in the dry weight of the pods, while treatment B35F140 increased the pod dry weight per plant by 9.94% and 117.49% in comparison to B70F140 and CK, respectively. When pod dry weight in treatments B35F70 and B70F70 was compared, treatment B35F70 showed a 32.66% increase over treatment B70F70. In the CK, the lowest pod numbers and pod dry weights was found.

**Table 5:** Average components of soybean yield in combination of biochar and organic fertilizer.

Treatments	Soybean Pods (Number /Plant)	Pod Dry Weight (g/Plant)	Soybean Seeds (Number /Plant)	Soybean Seeds Weight (g/Plant)
CK	11.33^a^	7.83^a^	23.00^a^	5.37^a^
B35F70	17.67^bc^	13.73^c^	38.00^bcd^	9.01^cd^
B35F105	18.00^bcd^	13.87^c^	36.17^bc^	8.26^bc^
B35F140	23.33^e^	17.03^d^	45.00^d^	10.77^d^
B70F70	15.00^b^	10.35^b^	31.67^b^	6.62^ab^
B70F105	20.00^cd^	14.55^c^	38.67^bcd^	9.44^cd^
B70F140	21.00^de^	15.49^cd^	40.33^cd^	9.91^cd^

Means with the same superscript lowercase letter within the column do not significantly differ at (*p< 0*.*05*) according to the least significant difference (LSD). CK represents un-amended soil. Numbers preceding letters B and F represent grams of applied biochar and organic fertilizer, respectively.

An increase in the number of soybean seeds per plant was also witnessed following the amendment of strongly acidic soil. In comparison to B70F70 and CK, treatment B35F140 resulted in an increase in the number of seeds per plant by 42.09% and 95.65%, respectively. However, when treatment B35F70 was applied, the number of seeds per plant did not statistically differ from treatment B70F105.

Only treatment B70F70 produced the fewest soybean seeds per plant among the soils amended with different rates of a combination of biochar and organic fertilizer. In contrast to the results regarding pod number, pod dry weight, and seed number per plant, the application of treatment B70F70 produced insignificant differences in terms of soybean seeds per plant when compared to CK. Moreover, treatment B35F140 produced more soybean seeds per plant by 62.69% and 100.56% in comparison with treatments B70F70 and CK, respectively.

### Linear regression relationship

**Fig 6:**
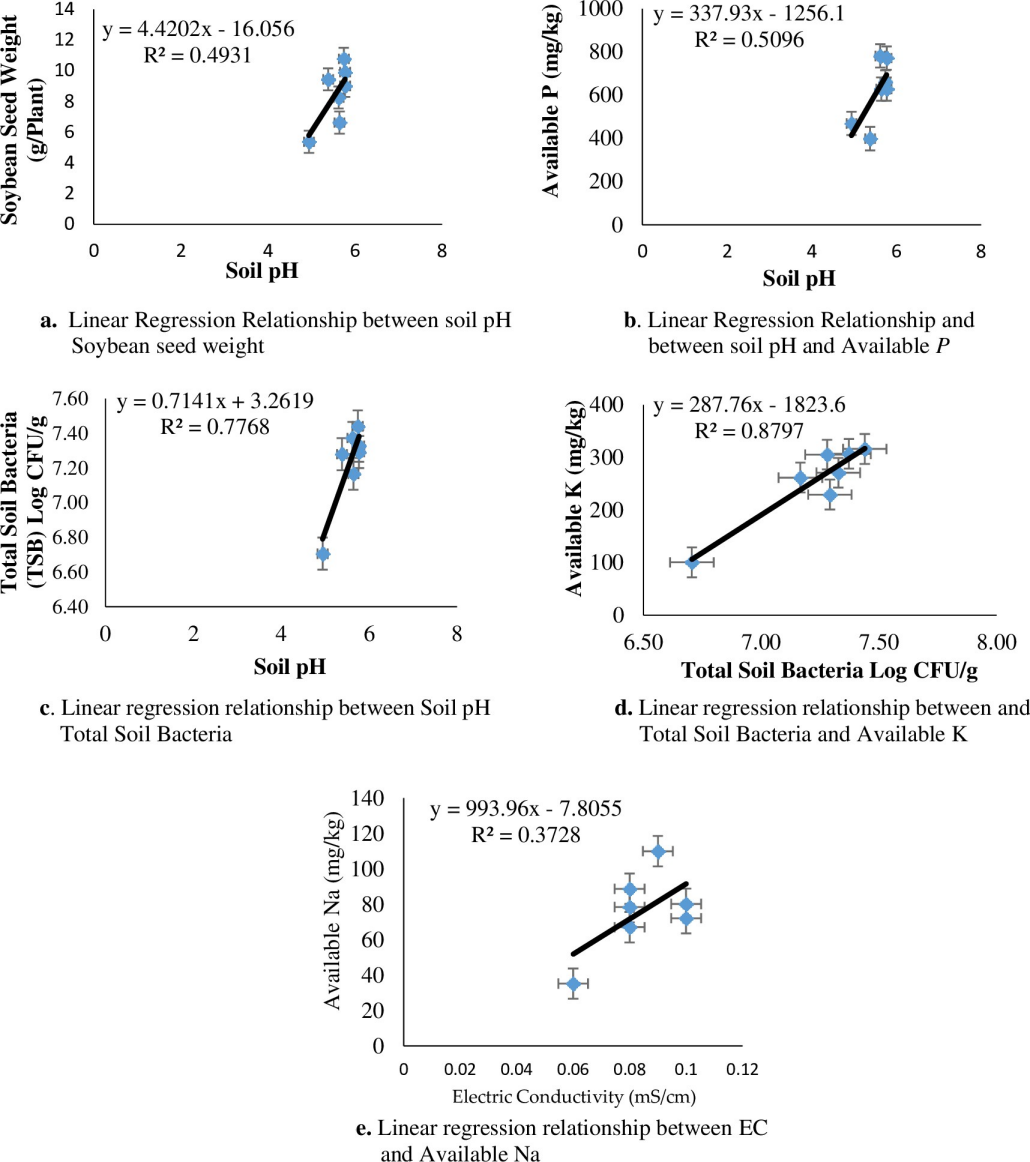
Linear regression relationship between soil properties and soybean traits.

There was a linear regression relationship between soil pH and soybean yield (R^2^ = 0.4931), available P (R^2^ = 0.5096), and total soil bacteria (R^2^ = 0.7768), as shown in Fig 6A–6C, respectively. This relationship indicates that an increase in soil pH in amended soils is correlated with an increase in soybean yield, available P, and population of bacteria in the soil. Moreover, as depicted in Fig 6D, we observed a strong linear regression relationship between available K and total soil bacteria (R^2^ = 0.8797), which indicated that a high concentration of available K in the soil was associated with an increase in soil bacteria proliferation. Fig 6E shows a linear regression relationship (R^2^ = 0.3728) between available Na and soil electrical conductivity.

## Discussions

### Effects of biochar and organic fertilizer on changes of soil pH, and electrical conductivity

The change in soil pH which occurred after the amendment of strongly acidic soil proves the ability of biochar and organic fertilizer to ameliorate soil acidity. Definitely, an increase in soil pH in amended soils was attributed to a biochar pH of 9.89 (alkaline). In addition, the pyrolysis process covers the surface of biochar by enormous carbonates and hydroxyl groups. As a result, biochar enhanced the sorption of cations, notably, H^+^, Fe^3+^, and Al^3+^ [48], which are abundant in strongly acidic soils. Furthermore, the pH of organic fertilizer was 8.22 (alkaline); therefore, the effects of alkaline on soil acidity were numerous. For this reason, strongly acidic soil was ameliorated to moderate acidity. Our findings on soil pH improvements are consistent with those of [33,49,50]. On the contrary, soil pH in CK decreased by 4.26% (Table 2) compared to a soil pH of 5.13 before soybean planting. This might have been due to the production of root exudates and decomposition of organic matter in experimental soil, which mainly produce the organic acids. As a result, there was a further decrease in soil pH in CK soils. Our findings in soil electrical conductivity refute those of [50]. Comparing the soil added with rice husk biochar only to the control soil group, they reported no significant difference in electrical conductivity. Our results explain the significant increase in EC in the amended soils as a result of the application of organic fertilizer. The electrical conductivity (EC) of soil is a measure that determines its salt content. It represents the concentration of ions or salinity. Furthermore, EC is an important indicator of soil health, soil microbial activity and nutrient availability. When the soil solution has a high concentration of soluble salts (>2.5 mS/cm), reverse osmosis pressure is generated, replacing the water in the root system and causing the root tip to dry or become brown.

### Effects of biochar and organic fertilizer on changes of soil organic matter content

Although treatment B35F140 resulted in the lowest content of organic matter (Table 2), a comparable treatment led to the largest number of soil bacteria (Fig 4). This infers that a large population of soil bacteria caused the rapid breakdown or mineralization of organic materials, mostly the organic matter contained in organic fertilizer, into soil-available nutrients. For this reason, soil organic matter significantly decreased compared to other treatments. On the other hand, this might be explained as the positive effects of a combination of biochar and organic fertilizer on soil bacteria expansion that led to successful mineralization. Moreover, treatment B35F140, which also raised the availability of potassium and zinc (Tables 2 and 3 respectively), provides additional evidence that an increase in soil bacteria population following amendment of strongly acidic soil by using biochar and organic fertilizer contributed to fruitful mineralization.

Furthermore, our study found an increase in organic matter content of 45.38%, 62.72%, and 63.29% in treatments B70F105, B70F140, and B35F105, respectively, compared to CK. This was apparently attributed to the organic amendments, particularly the organic fertilizer, which is composed of cattle, chicken, and pig manures, sawdust, and soybean crop residues. Our findings agree with those of [51], who found that, application of cattle manures significantly improved soil organic matter. Organic matter in the soil improves its capacity to retain water and nutrients while also increasing their availability. It also encourages soil aggregation.

### Changes in soil available P, K, Ca, Mg, Cu, Zn, Fe, Mn, and Na

Our findings on soil available phosphorous (P) are comparable with those of [52–55], who discovered that biochar application improved available P in agricultural soils. Because biochar has a liming effect once it alters the soil acidity to moderate acidity, available P is free in soil medium. Furthermore, biochar has the advantageous attribute of having multiple pores on its surface. This feature enhances nutrients adsorption including phosphorous on the biochar surface, hence lessening *P-fixation* by iron oxides. As a result, phosphorous is being available in soil for plant uptake. Among the amended soils, treatment B70F105 showed the lowest quantity of available P in the soil. However, with a comparable treatment, the soil pH remained strongly acidic (5.38). This could result in the *P-sorption* due to *P-fixation* by either Al^3+^ or Fe^3+^. Typically, plants absorb phosphorus from the soil as inorganic phosphate (Pi). Once the *Pi* reaches in the plant, is transformed into organic forms such as adenosine triphosphate (ATP) and adenosine diphosphate (ADP), which are important molecules in energy transmission during the process of photosynthesis [55].

The combination of biochar and organic fertilizer, together with strongly acidic soil, significantly increased potassium, as evidenced in the results (Table 3). When organic manure is decomposed by the soil microbes, organic acids, notably *humic* and *fluvic acids*, are produced [56]. The afore-mentioned two organic acids aid in the dissolution of minerals in soils, particularly those containing potassium [57]. Moreover, it has been reported that humic acids addressed the disturbance in physio-biochemical status produced with stresses, maintaining the crop productivity [58–60]. In these mechanisms, potassium ions were produced throughout the dissolving process, and increased the potassium content in the soil. Furthermore, the applied biochar contained potassium nutrients, and when the soil bacteria decomposed organic fertilizer, the soil was also supplemented with available potassium from organic fertilizer. This significantly increased the available potassium in the amended soil. This increase in available potassium in amended soils is consistent with findings of [61–63]. Potassium plays a key function in stomata-opening regulation, allowing for appropriate or inadequate or inadequate gas and water fluxes in plants depending on its concentration in plant cells [64,65].

Our study found that using a combination of biochar and organic fertilizer resulted in a significant increase in available calcium and magnesium (Table 2). Calcium is an essential mineral for plants. It contributes significantly to the development of the plant cell wall membrane, protecting cell integrity and membrane permeability [66]. Magnesium is also documented as an essential plant nutrients, it involves in chlorophyll synthesis [67], activation of enzymes such as *Rubisco*, *ATPase*, and *Protein kinase* [68]. In addition, it participates in the synthesis of proteins. Our findings on the significant increase in accessible Ca and Mg are consistent with [69].

Soybean plants require a small quantity of the trace elements, among them are copper, zinc, iron, manganese, and sodium for optimal growth and yield formation. The absence of a sufficient quantity of these trace elements in soils can result in a notable negative effects on crop growth and productivity. Iron plays a significant role in various physiological and biochemical pathways in plants. In addition, it serves as a component of many vital enzymes, such as cytochromes of the electron transport chain. In plants, iron is involved in the synthesis of chlorophyll and is essential for the maintenance of chloroplast structure and function [70]. Manganese plays a role as cofactor for the oxygen-evolving complex of the photosynthetic machinery. Moreover, it involves in catalyzing the reaction during the split of water in photosystem II (PSII) [71]. On the other hand, Na^+^ and K^+^ have been well known as energetic cations in transportation across the cell membranes. However, the only example of a Na^+^ driven cotransport system recognized in plants so far is a pyruvate transporter localized at the chloroplast envelope membrane [72]. Copper (Cu) is a cofactor for a variety of enzymes, and it plays an important role in photosynthesis, respiration, the antioxidant system, and signal transduction [73]. Plants require zinc (Zn) mostly for increasing the production of cytochrome, regulating auxin hormone, restoring photosystem II, stabilising CO_2_ in mesophyll, and biosynthesizing carbohydrates [74]. The combination of biochar and organic fertilizer led to a significant enhancement in the available copper and zinc in the soil. This was due to the mineralization of the organic fertilizer carried by the soil microorganisms, particularly the bacteria. Studies of [75,76] have indicated that the application of organic fertilizer could enhance the availability of copper and zinc nutrients in soils.

Overall, amendment of strongly acidic soil by using a combination of biochar and organic fertilizer resulted in a significant increase of the ascertained soil nutrients. This was a result of the organic materials added to strongly acid soil: biochar and organic fertilizer. Since all nutrients, N, P, K, Ca, Mg, Cu, Zn, Fe, Mn, and Na make up the biomass of organic residues, thus when organic fertilizer was decomposed by soil bacteria all these nutrients were released into the soil [37]. Moreover, significant improvement in soil nutrients is due to general fertility improvement mechanism [35].

### Response of soil bacteria proliferation to application of biochar and organic fertilizer

Amendment of strongly acid soil by using organic components changed soil pH from strongly acid to moderately acid in the five applied treatments (Table 2). As a result, it encouraged the growth of soil bacteria (Fig 4). Furthermore, the organic matter content (61% of organic fertilizer) provided an essential source of food for soil microbes; as a result, soil bacteria proliferated several times. Biochar, on the other hand, has shown to provide an important habitat for soil microbes. This is due to the abundance of pores formed on its surface after the pyrolysis process [77]. The pores allow microorganisms to find safe sanctuary and flourish, while also safeguarding them from other predators in the soil. Bacteria have an important role in decomposition, notably organic matters contained in the organic fertilizers. The activity changes inaccessible soil nutrients into readily available forms that may be taken by plants roots. Our results on total soil bacteria improvement align with previous research [43,78]. Conversely, the lowest quantity of soil bacteria that was found in the CK treatment could be due to the fact that the soil pH was further lowered to very strongly acid (4.94). As a result, the growth of bacteria in the CK soil group was limited.

### Effects of combination of biochar and organic fertilizer on the SPAD values

The SPAD values indicate the relative amount of leaf chlorophyll, which is crucial for photosynthesis. Conversely, the SPAD values reflect the nitrogen content of plant leaves. Nitrogen is the most plant growth-limiting nutrient and plays a role in yield impact as well as the synthesis of protein in soybeans. The enhancements in chlorophyll content in our study are in agreement with [43]. However, soybean is a legume crop that can fix atmospheric nitrogen into the forms that are available in soil for plant root uptake. But when we compared our results between the amended soils and the CK treatment, soybean plants grown in the latter treatment significantly had the lowest content of chlorophyll. This implies that, under strongly acidic conditions, soybean (*Glycine max* L.) performance on nitrogen fixation was either limited or its roots were not able to access a sufficient amount of available soil nitrogen. However, the added biochar and organic fertilizer each contained some amount of nitrogen. Therefore, following the increase in soil pH, the soybean roots extensively grew to absorb a sufficient amount of available nutrients, including nitrogen from the soil. Moreover, [63] reported increased biological nitrogen fixation in soybeans following biochar application.

### Response of soybean root nodule to application of biochar and organic fertilizer

The average number of soybean root nodules per plant in each treatment was counted after harvest. Although our root nodule results were not significantly different among the treatments, our findings agree with those of [46,63], who found that application of biochar increased the number of root nodules as opposed to no biochar application. Root nodules are important structures formed in the roots of legume crops, they are special as bacterial habitats that assist in the fixation of atmospheric nitrogen into soil available nitrogen.

### Soybean yield components response to application of biochar and organic fertilizer

Treatment B70F70 was among the amended soils that showed a low response to the components of soybean yield. Moreover, in a similar treatment, only the results of soybean seed weight did not significantly differ when compared to the CK treatment. However, our findings on soil pH, available P, and chlorophyll content in the application of such treatment were the lowest as compared with other treatments among the amended soils. This might be the reason why such treatment resulted in no significant seed weight as compared to CK treatment. Furthermore, the application of treatment B35F70 significantly increased soybean seed weight per plant compared to B70F70 (Table 5). The increase was 36.10%. However, treatment B35F70, among the treatments showed the highest increase in soil pH, available P (Table 2), and the development of root nodules (Fig 5) after amendment of strongly acidic soil. For these reasons, it is obvious that our findings in treatment B35F70 significantly differed from those of B70F70. Our findings on improvements in soybean yield agree with those of [44,63]. Overall, a combination of biochar and organic fertilizer showed a positive effect on our research findings. This was triggered by a change in soil quality from strongly acidic to moderately acidic. This change permitted vital plant nutrients to be available in the amended soils. As a result, when soybeans were planted in the amended soils, root hairs extensively grew. Therefore, compared to unamended soils, soybean plant roots absorbed a sufficient amount of available nutrients and water from the amended soils.

## Conclusion

We conclude that after amendment of strongly acidic soil by using a combination of biochar together with organic fertilizer, the ascertained soil physico-chemical properties significantly improved. Soil pH, which is the master of soil chemical and biological processes, altered from strongly to moderately acid. The moderately acid condition is suitable for plant growth, nutrient availability, and microbial proliferation in soils. Furthermore, there was significant enhancement on total soil bacteria population in the amended soils. In addition, our study found that a combination of biochar and organic fertilizer greatly increased leaf chlorophyll content compared to soybean plants cultivated under strongly acidic soils. Moreover, when soybean plants were cultivated on the amended soils, we found more root nodule development than in the control soil group. Overall, the best response to the available potassium and zinc, soil electrical conductivity, bacteria proliferation, chlorophyll content, and soybean pod number, pod dry weight, number soybean seeds per plant, and seed weight per plant were obtained through the application rates of biochar (35 g/pot) and organic fertilizer (140 g/pot). Consequently, it’s evident that a combination of biochar and organic fertilizer could improve the soil physical, chemical, biological properties, and agronomic attributes of soybean crop under strongly acidic soil. However, because our study only focused on one season and utilized pot experiment, we also recommend additional research on the impacts of a combination of biochar and organic fertilizer at different soybean growing seasons and in a field environment. Furthermore, we recommend more research on the quality of soybean protein in the seeds when they are grown by using a combination of biochar and organic fertilizer. Finally, many biochar research has not addressed the economic impacts of applying biochar in crops production, therefore, there is another gap of knowledge which needs to be filled by assessing economic impacts due to application of biochar and organic fertilizer on soybean production.

## Supporting information

S1 Data (XLSX)
