# Correction: Effects of the combination of biochar and organic fertilizer on soil properties and agronomic attributes of soybean (*Glycine max* L.)

**DOI:** 10.1371/journal.pone.0324843

**Published:** 2025-05-20

**Authors:** Marianus Evarist Ngui, Yong-Hong Lin, I-Lang Wei, Chia-Chung Wang, Ya-Zhen Xu, Ying-Hong Lin

There are errors in the author affiliations. The correct affiliations are as follows:

Marianus Evarist Ngui, Yong-Hong Lin, I-Lang Wei, Chia-Chung Wang, Ya-Zhen Xu and Ying-Hong Lin

**1** Department of Tropical Agriculture and International Cooperation, National Pingtung University of Science and Technology, Taiwan. **2** Soil and Fertilizer Laboratory, Department of Plant Industry, National Pingtung University of Science and Technology, Taiwan **3** Molecular Plant Medicine Laboratory, Department of Plant Medicine, National Pingtung University of Science and Technology, Taiwan.

In the Abstract section, there is an error in the second sentence. The correct sentence is: A greenhouse and pot experiment was designed using a randomized complete block design with factorial 2 × 3 treatments and three replications.

In the Abstract section, there is an error in the sixth sentence. The correct sentence is: ‘In addition, the amendment of strongly acidic soils with a combination of biochar and organic fertilizer resulted to a significant increase in soil organic matter, available phosphorus (P), potassium (K), calcium (Ca), magnesium (Mg), iron (Fe), copper (Cu), manganese (Mn), zinc (Zn), and sodium (Na) compared to the control group (CK).

In the Soil, biochar, and organic fertilizer analysis subsection of the Materials and methods, there is an error in the first sentence. The correct sentence is: A composite soil sample (0–20 cm depth) was collected prior to amendment for the purpose of analyzing its physical, chemical, and biological characteristics.

In the Soil, biochar, and organic fertilizer analysis subsection of the Materials and methods, there is an error in the first sentence. The correct sentence is: The physico-chemical properties of the organic fertilizer were as follows: pH 8.22, organic matter 61%, total N 2.5%, P 2.3%, and K, 2.0%.

In the Experimental duration and design subsection of the Materials and methods, there is an error in the second sentence. The correct sentence is: The study utilized a randomized complete block design (RCBD) with factorial 2 × 3 treatments, replicated three times, using pots as experimental units.

In the Data sampling and collection subsection of the Materials and methods, there is an error in the first sentence. The correct sentence is: To estimate the relative leaf chlorophyll content, a portable chlorophyll meter, SPAD-502 (Konica-Minolta, Tokyo), was utilized.

There is an error in the caption of Table 3. Please see the correct Table 3 here.

In the Soil available P, K, Ca, Mg, Cu, Zn, Fe, Mn, and Na subsection of the Results, the fifth sentence of the second paragraph should not have been included.

In the Soil available P, K, Ca, Mg, Cu, Zn, Fe, Mn, and Na subsection of the Results, there is an error in the seventh sentence. The correct sentence is: After 100 days of amending strongly acidic soil, the available copper concentration significantly increased in treatments B70F70 and B70F105 compared to treatments CK, B35F70, and B35F105 (Table 3).

**Table 3:** Soil available Fe, Cu, Mn, Zn, and Na after 100 days of amendment of strongly acidic soil by using a combination of biochar and organic fertilizer.

Treatments	Available trace elements				
	Fe (mg/kg)	Cu (mg/kg)	Mn (mg/kg)	Zn (mg/kg)	Na (mg/kg)
CK	181.02^ab^	0.87^a^	2.54^a^	4.74^a^	35.17^a^
B35F70	219.47^bc^	1.05^a^	4.87^ab^	7.44^bc^	88.76^c^
B35F105	246.07^c^	0.97^a^	6.95^bc^	9.20^c^	67.09^b^
B35F140	170.65^a^	1.25^ab^	5.43^abc^	9.35^c^	72.15^bc^
B70F70	221.47^bc^	1.94^b^	6.26^bc^	8.35^bc^	110.01^d^
B70F105	200.40^ab^	1.88^b^	8.37^c^	8.95^c^	80.19^bc^
B70F140	180.35^ab^	1.28^ab^	2.25^a^	6.14^ab^	78.43^bc^

Means with the same superscript lowercase letter within the column do not significantly differ at (*p < 0.05*) according to the least significant difference (LSD). CK represents unamended soil. Numbers preceding letters B and F represent grams of applied biochar and organic fertilizer, respectively.

In the Total Population of Soil Bacteria subsection of the Results, the last sentence of the paragraph is missing. The correct sentence is: However, only the bacterial colonies were considered in our observations.

There is an error in the panel A of Fig 6. Please see the correct Fig 6 here.

**Fig 6:**
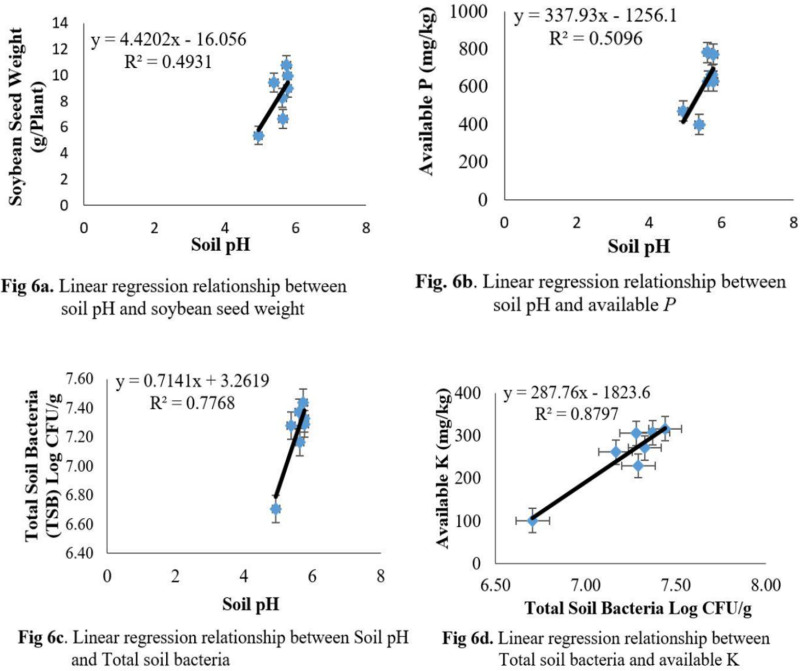
Linear regression relationship between soil properties and soybean traits.

There are grammatical errors in the Conclusion section. The correct Conclusion section is: Our study concludes that amending strongly acidic soil with a combination of biochar and organic fertilizer significantly improved the evaluated parameters in this study. Soil pH, a critical factor governing soil chemical and biological processes, changed from strongly acidic to moderately acidic. This moderate acidity is more conducive to plant growth, nutrient availability, and microbial proliferation. Additionally, there was a significant increase in the total soil bacteria population in the amended soils. Our findings also revealed that soybean plants grown in the amended soils exhibited greater leaf chlorophyll content compared to those cultivated in unamended, strongly acidic soils. Moreover, root nodule development was more pronounced in soybean plants grown in the amended soils than in the control group. The most favorable responses in key parameters—including available potassium and zinc, soil electrical conductivity, bacterial proliferation, chlorophyll content, and soybean yield attributes such as pod number, pod dry weight, number of seeds per plant, and seed weight per plant—were achieved with application rates of 35 g of biochar per pot and 140 g of organic fertilizer per pot. Therefore, combining biochar and organic fertilizer can enhance the physical, chemical, and biological properties of strongly acidic soil while improving the agronomic performance of soybean crop. However, as our study was limited to one growing season and conducted under controlled pot conditions, further research is needed to evaluate the long-term effects of biochar and organic fertilizer under various soybean growing seasons and in field environments. We also recommend additional studies on the impact of biochar and organic fertilizer on the quality of soybean protein and oil content. Furthermore, there is a notable knowledge gap regarding the economic implications of using biochar in crop production. Future research should assess the cost-effectiveness and economic benefits of applying biochar and organic fertilizer in soybean cultivation.

## References

[1] Ngui ME, Lin Y-H, Wei I-L, Wang C-C, Xu Y-Z, Lin Y-H. Effects of the combination of biochar and organic fertilizer on soil properties and agronomic attributes of soybean (*Glycine max* L.). PLoS One. 2024;19(9):e0310221. doi: 10.1371/journal.pone.0310221 39298498 PMC11412508

