# Correction: Correction: Effects of the combination of biochar and organic fertilizer on soil properties and agronomic attributes of soybean (*Glycine max* L.)

**DOI:** 10.1371/journal.pone.0336585

**Published:** 2025-11-10

**Authors:** Marianus Evarist Ngui, Yong-Hong Lin, I-Lang Wei, Chia-Chung Wang, Ya-Zhen Xu, Ying-Hong Lin

S1 Data is incorrect. Please view the correct S1 Data below.

## Supporting information

S1 Data (XLSX)
